# Cases Illustrating the Efficacy of Arsenic in Intermittent Fever

**Published:** 1827-11

**Authors:** William Teevan

**Affiliations:** Member of the Royal College of Surgeons, and Licentiate of the Company of Apothecaries of London.


### TREATMENT OF INTERMITTENTS.

*Cases illustrating the Efficacy of Arsenic in Intermittent Fever.* By Mr. William Teevan, Member of the Royal College of Surgeons, and Licentiate of the Company of Apothecaries of London.

Were it necessary, I could adduce numerous cases which have been treated during the present season, in proof of the very great value of arsenic in intermittent fever, although this medicine appears to have fallen into unmerited disuse. It will be observed, that in Case the IIId. in which the sulphate of quinine, and in Case the Vth. the bark, were given without any material advantage, the paroxysms were speedily arrested by the arsenic. Should cough or sickness of the stomach supervene during the administration of this medicine, it ought to be relinquished immediately, until these symptoms are subdued, which should be combated by the usual remedies had recourse to on other occasions.

Case I.—A. B., forty-six years of age, states that he was attacked with ague six months ago, which came on every morning at eight o'clock, and continued the greater part of the day. He complains of pain in the head and loss of appetite; tongue covered with a white fur.

March 7th, 1827.—℞. Infus. Sennæ ℥jss.; Magnes. Sulph. ʒijss.; Syr. Simplicis ʒij. M. fiat haustus statim sumend.—℞. Liq. Arsenicalis m. x.; Aq. distillatæ ℥jss. M. fiat haustus post operat. haust. purg. sumendus, et quartis horis repetend.

9th.—No return of fever; tongue clean; bowels regular; pains in the bones.

12th.—Quite well; has had no return of fever since the commencement of the medicine.

Case II.—R. S., twenty-four years of age, came under my care, having ague of tertian type, which has existed seven weeks. The paroxysms come on at irregular intervals, so that he could not calculate the time of their approach.

March 10th, 1827.—℞. Haust. Arsenical ut supra præscrip. quartis horis sumendus.

12th.—He had a slight attack of fever yesterday, which did not last more than two hours.

Perstat in usu medicament.

14th.—The ague has not returned since the last report.

17th.—Remains quite well; discharged cured.

Case III.—James Roberts, thirty-two years of age, says that, three months ago, he was taken ill of ague, which came on regularly at eight o'clock a.m.: latterly, however, the paroxysms came on every other day at four o'clock p.m. and continued throughout the night. He has taken the sulphate of quinine upwards of three weeks, with temporary advantage; the paroxysms returning as soon as he left off the medicine.

May 24th, 1827.—℞. Liq. Arsenicalis m. x.; Aq. distillatæ ℥jss. M. fiat haustus quartis horis sumendus.

26th.—The ague has not returned since he took the medicine; expresses himself much relieved.

Perstat in usu medicament.

28th.—Quite well.

Case IV.—Alexander Reilly, ætat. forty-three, states that he has been labouring under ague three weeks, and has not taken any medicine. The paroxysms make their appearance every other day, at irregular periods, and generally last for eight or ten hours.

April 17th, 1827.—℞. Liq. Arsenicalis m. x.; Aq. distillatæ ℥jss. M. fiat haustus quartis horis sumend.

20th.—Better: has had no return of fever.

Repet$^r$ medicament.

24th.—Quite well; discharged cured.

Case V.—A. Robinson, ætat. thirty-seven, eleven weeks ill of quartan ague, says he has been two months in hospital, and has taken bark without deriving any advantage; tongue white; appetite impaired; bowels irregular; sense of heaviness in the head; and soreness over the body, as if it had been bruised; pain in the bones.

June 2d, 1827.—℞. Haust. Sennæ c. Magnes. Sulph. statim sumendus. ℞. Liq. Arsenicalis m. xv.; Aq. distillatæ ℥jss. M. fiat haust. quartis horis sumend. post operat. haust. purg.

5th.—Fever at the usual period; great sickness of stomach after taking the medicine; tongue white; bowels confined; pain in the bones.

Repet$^r$ Haustus Purg. stat. sumend., et omitt$^r$ Liq. Arsenical.

6th.—Bowels open three or four times; tongue clean; appetite improved.

Repet$^r$ Liquor Arsenicalis quartis horis.

10th. No return of fever.—14th. Quite well; discharged cured.

Case VI.—Sarah Brady, aged twenty-seven, ten days ill of ague, tertian type; she has taken no medicine.

June 4th, 1827.—℞. Haust. Liq. Arsenical. ut supra præscrip. quartis horis sumend.

7th.—No return of the paroxysms.

Perstat. in usu medicament.

10th.—Having remained well since the last report, she was discharged.

[The doses of arsenic employed by Mr. Teevan in the above cases were larger than those usually given: perhaps, however, we are in general rather timid in the use of powerful remedies. In a recent Number of the New-York Medical and Physical Journal, a case of Chorea is detailed by Dr. James Fountain, in which a young lady took ten drops of the Liq. Arsenalis every two hours for four days: the pulse was raised to 120; and the vascular action became "intense;" the patient, however, speedily recovered.]

---

